# Promoting Learning About Nutrition and Healthy Eating Behaviors in Chinese Children Through an Alternate Reality Game: A Pilot Study

**DOI:** 10.3390/nu17071219

**Published:** 2025-03-30

**Authors:** Ruobing Wang, Jie Yao, Claudia Leong, Elena Moltchanova, Simon Hoermann

**Affiliations:** 1 School of Product Design, University of Canterbury, 20 Kirkwood Ave., Upper Riccarton, Christchurch 8041, New Zealand; ruobing.wang@pg.canterbury.ac.nz; 2 School of Economics and Management, Harbin Institute of Technology Shenzhen, Pingshan First Road, Shenzhen 518067, China; yaojiejulie@hit.edu.cn; 3 Department of Applied Science and Social Practice, Ara Institute of Canterbury, Madras Street, Christchurch Central City, Christchurch 8041, New Zealand; claudia.leong@ara.ac.nz; 4 School of Mathematics and Statistics, University of Canterbury, Private Bag 4800, Christchurch 8140, New Zealand; elena.moltchanova@canterbury.ac.nz

**Keywords:** nutrition education, dietary behavior change, Chinese children, alternate reality game, gamified learning, school-based nutrition intervention

## Abstract

**Background**: Childhood obesity is a growing public-health concern in China and globally, a trend influenced by multiple factors, including poor eating behaviors and insufficient physical activity. While interactive health games have shown promise in improving children’s nutrition education and healthy eating behaviors, few have been tailored for the Chinese context. This study aimed to develop and evaluate Happy Farm, Happy Meal (HFHM), an alternate reality game (ARG) integrated into Chinese elementary students’ daily routines to enhance their nutrition knowledge and improve their eating behaviors. **Methods**: This pilot study employed a quasi-experimental design with two third-grade classes, which were randomly assigned to the HFHM intervention group (*n* = 40) or a no-game control group (*n* = 39). The game design was informed by a pre-intervention survey and interviews with caregivers and teachers, which identified key dietary challenges such as picky eating, slow eating, and food waste. Over a two-week period, the HFHM group engaged in food- and nutrition-focused tasks that were incorporated into their lunchtime routines. Pre- and post-intervention data were collected on nutrition knowledge, food waste, picky eating, and meal duration, with daily progress tracking in the HFHM group. **Results**: Compared to the control group, the HFHM group showed a significant increase in nutrition knowledge (*p* < 0.05), reduced food waste (*p* < 0.01), decreased picky eating (*p* < 0.01), and improved meal duration (*p* < 0.05). However, the small sample size and short intervention period limit generalizability. **Conclusions**: These findings suggest HFHM is a promising tool for improving nutrition education and dietary behaviors in Chinese children. Future research should validate these findings in a larger sample and assess long-term impacts.

## 1. Introduction

Childhood obesity is increasingly recognized as a significant public-health challenge in China and worldwide [1,2]. According to the 2021 Children’s Blue Book and China Children’s Development Report [3], the prevalence of overweight and obesity among Chinese children aged 6 to 18 years was 24.2% in 2019 and increased to 29.4% in 2022. Overweight and obesity in childhood can increase the occurrence of physical and psychological diseases, including blood lipid disorders, hypertension, type 2 diabetes, cardiovascular disease, eating disorders, and other diseases. It has been demonstrated that unhealthy lifestyles are one of the main causes of obesity and overweight in childhood. Insufficient physical activity, high consumption of fatty, sweet, and processed food, low consumption of fruit and vegetables, and unhealthy eating behaviors are believed to be the most significant lifestyle factors that increase the risk of obesity and overweight in children and adolescents [4,5,6]. Therefore, the prevention of obesity and overweight, especially in childhood, involves informing children and families about nutrition, physical activity, and healthy lifestyle behaviors [7]. However, among Chinese children, various eating behavior problems, such as poor appetite, bad eating habits, special preference for certain foods, and fear of eating, are prevalent [8]. Insufficient nutrition knowledge also exacerbates the issue, creating a significant barrier to obesity prevention [9]. Despite the fact that the Dietary Guidelines for Chinese Residents [10] have been integrated into the curriculum of primary and secondary schools in China since 2016, a survey revealed that only 10% of students possess relevant nutrition knowledge [11].

Traditional education methods, though informative, often fail to foster behavioral change due to their limited interactivity and nonuse of experiential learning opportunities [12,13]. Therefore, it is important to design more engaging and effective tools for nutrition education and the establishment of healthy eating behaviors in childhood. In this context, serious games have gained attention as a promising approach in recent years [14]. Multiple studies have demonstrated the effectiveness of serious games at improving children’s nutrition knowledge and eating behaviors [15,16,17,18]. With the potential to encourage participation and motivation in learning activities, game-based interventions can promote more effective learning among elementary-school children [19]. However, as serious games require the use of electronic devices, whose usage is strictly regulated by schools and parents in China, their widespread adoption faces hindrances and challenges [20].

Alternate Reality Games (ARGs), which blend real-life activities with gamified storytelling, could be a better choice for Chinese school settings, as they are less dependent on technology [21]. Research indicates that ARGs can enhance educational experiences by encouraging students to engage actively with the material through collective problem-solving and narrative exploration [22,23,24]. In addition, studies have also shown that the participatory nature of ARGs allows children to take ownership of their learning [25], making health education more relatable and impactful. Currently, ARGs have been applied in fields like language and culture education, science education, and physical education. However, there are few evidence-based ARGs whose purpose is to support nutrition education [26].

Public primary schools in China provide an ideal environment in which to implement programs for nutrition education and improving eating behaviors. Integrating nutrition education into regular school activities has been shown to improve not only children’s dietary behaviors but also those of their families [27,28]. Although research supports the benefits of nutrition education, the application of gamification in this field remains unexplored in Chinese elementary schools [29].

Thus, this study was conducted to address these research gaps. The ARG Happy Farm, Happy Meal (HFHM) was developed to enhance Chinese primary-school children’s nutrition knowledge and improve their eating behaviors. The game design was informed by a pre-intervention survey and interviews with caregivers and teachers from the participating school, which identified three major eating-behavior challenges among the children: pickiness, food waste, and slow eating. These findings shaped the core focus of the game and intervention. To enhance experiential learning, the participating school established a garden near the campus, with each class allocated a specific planting area. This feature was incorporated into the game design to connect virtual gameplay with real-world gardening experiences. Grounded in self-determination theory (SDT) [30], HFHM employs game mechanics such as competition, cooperation, goals, and rewards to fulfill children’s psychological needs for autonomy, competence, and relatedness, which are essential for fostering intrinsic motivation and sustaining behavior change [31].

The primary objective of this study was to evaluate the efficacy of HFHM in promoting children’s nutrition knowledge and improving their eating behaviors. Specifically, we hypothesized that participation in HFHM would lead to significant improvements in children’s understanding of nutrition concepts, as well as to reductions in behaviors related to picky eating, food waste, and slow eating.

## 2. Materials and Methods

### 2.1. Pre-Intervention: Study Design and Participants

To ensure the game design was both effective and relevant to the target audience, preliminary research was conducted in May 2021. This involved administering a validated dietary-behavior questionnaire to 79 caregivers of children from the participating school. Among the returned questionnaires, 79 were valid for basic demographic information, while 38 were valid for assessing children’s dietary behaviors. The questionnaire, the Chinese School-aged Children’s Eating Behavior Scale, was developed and preliminarily evaluated by Zhang et al. [5] and has been shown to reliably assess dietary behaviors in school-aged children in China. To gain a better understanding of the participants, we included questions about their demographics, such as the caregiver’s education level and annual income. In addition, semi-structured interviews were conducted with five caregivers and two teachers to gain a deeper understanding of the children’s challenges related to dietary behaviors. The interview questions were designed to explore the contextual factors affecting children’s eating habits (Table 1).

### 2.2. Pilot Study: Intervention Design and Participants

Based on the pre-intervention survey, we established the objectives of the intervention: to enhance children’s nutrition knowledge and improve three eating behaviors, namely, pickiness, food waste, and slow eating. This study employed a between-groups design with two conditions: an experimental group and a control group. The participants were 79 children from two classes of the same grade in a primary school in Zhejiang Province, China. Each class was randomly assigned to one of the two conditions. In the experimental group, as the intervention, 40 children participated in the alternate reality game Happy Farm, Happy Meal (HFHM). The control group, consisting of 39 children, did not receive the intervention.

Prior to the intervention, parental consent was obtained after the study personnel had explained the research objectives and procedures. Children’s weights and heights were measured every semester by the school’s teachers to track their growth. With the consent of the school and parents, we obtained these data to calculate Body Mass Index (BMI). Then, each child’s health status was categorized as underweight, healthy weight, overweight, or obese based on standards established in the Criteria for Overweight and Obesity Screening Body Mass Index in Chinese School-Aged Children and Adolescents [32]. Additionally, demographic information was collected via questionnaires administered to both groups.

Data were collected in three distinct stages. Before the intervention, we assessed eating behaviors and levels of nutrition knowledge in both the experimental and control groups to establish a baseline. For the experimental group, daily data on eating behaviors and results of nutrition quizzes were collected during the intervention period. After the intervention concluded, eating behaviors and nutrition knowledge were reassessed in both groups. Due to logistical constraints, we were unable to collect daily process data from the control group, so only pre- and post-intervention assessments were conducted for this group.

**Figure 1:**
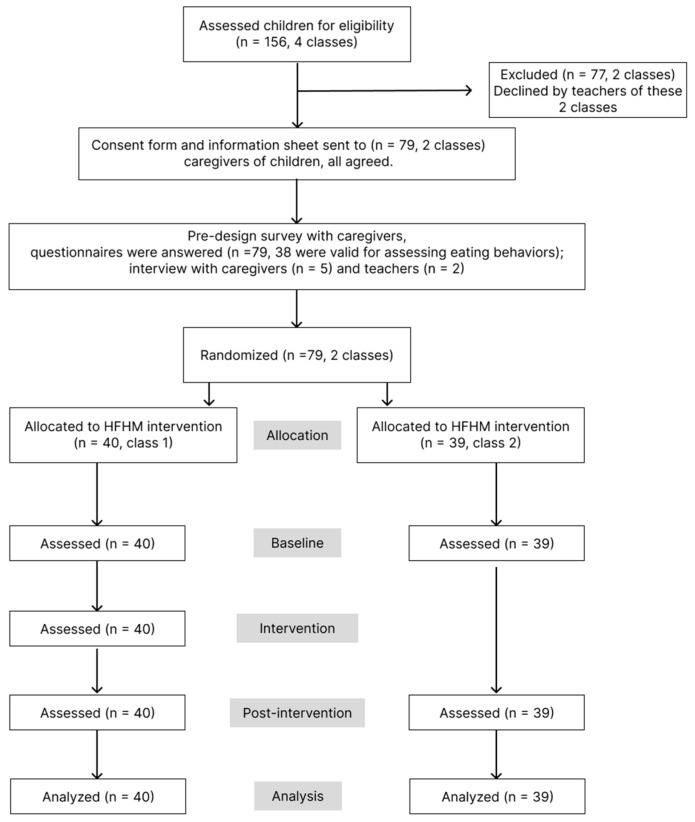
Flow chart of the pilot study.

The experiment was conducted during the 2021/2022 academic year, and the intervention occurred in November. The final data sample included 79 participants (Figure 1).

### 2.3. Measures

#### 2.3.1. Nutrition Knowledge

A questionnaire on nutrition knowledge was designed according to the Dietary Guidelines for Chinese Residents (2016) [10]. There were 20 questions, each worth one point. Table 2 shows some example questions.

#### 2.3.2. Eating Behaviors

Based on the children’s lunches at school, our study developed specific methods for evaluating three eating behaviors. In the experimental school (as in many other elementary schools in China), the lunchtime is limited to 25–30 min, which aligns with the recommended meal duration [33]. The school provides lunch daily, and the lunch consists of three dishes and rice. Each child was assigned the same types of food for every meal, but they could decide the portion sizes based on their appetite. However, children were not allowed to refuse any type of food, regardless of their preferences. To ensure the evaluation remained unbiased, the measurement criteria were not disclosed to the children. This prevented them from deliberately taking smaller portions during lunch distribution in an attempt to win the game. Three eating behaviors data were recorded according to the methods described below.

(1) Meal Duration
  Record the start of each child’s mealtime as T1 and the end of the mealtime as T2. The child’s meal duration is then calculated as follows:
  T0 = T2 − T1(1)
(2) Food Waste
  (a) Measure and record the weight of each child’s empty plate (G0).
  (b) Measure and record the combined weight of the food and the plate before lunch (G1).
  (c) Measure and record the combined weight of the leftover food and the plate after lunch (G2).
  (d) Calculate the food waste rate (FWR) using the following formula:

(2) $$FWR=\frac{G2-G0}{G1-G0}$$

**Figure 2:**
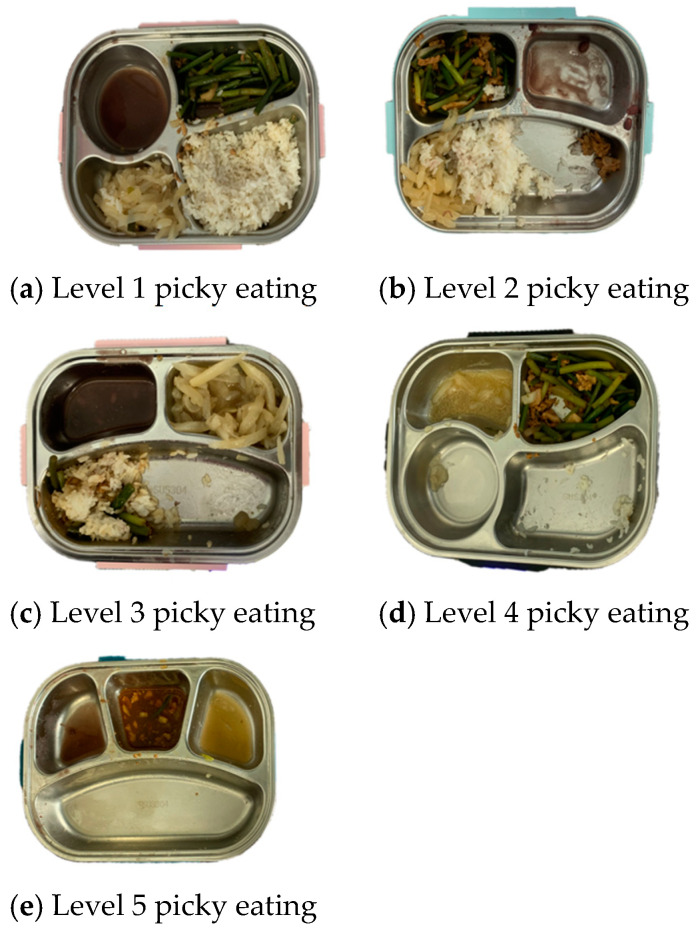
Example pictures showing different levels of picky eating. (**a**) Level 1; (**b**) Level 2; (**c**) Level 3; (**d**) Level 4; (**e**) Level 5.

(3) Picky Eating
  In collaboration with schoolteachers, a set of criteria was developed to assess the extent of children’s picky-eating behaviors. The evaluation considers both the types and amounts of food consumed and left uneaten by the children. The following is the rating scale for assessing picky eating:
  (a) Level 1: Severe picky eating (Figure 2a). Consumed one food item entirely, leaving the rest almost untouched.
  (b) Level 2: Moderate picky eating (Figure 2b). Consumed one type of food and partially consumed remaining items.
  (c) Level 3: General picky eating (Figure 2c). Consumed 2–3 types of food, with over half of the other items left uneaten.
  (d) Level 4: Mild picky eating (Figure 2d). Consumed 2–3 types of food, left only a small portion of the remaining items uneaten.
  (e) Level 5: No picky eating (Figure 2e). Consumed all food items or left a balanced portion of each item uneaten.

### 2.4. Game Design

**Figure 3:**
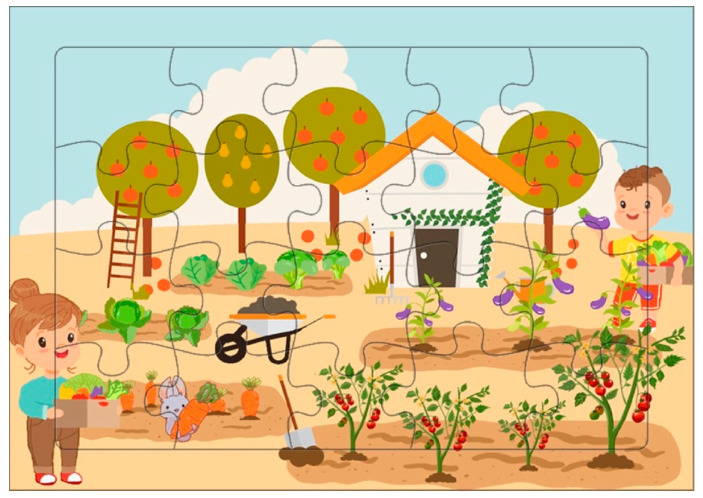
Puzzle designed for the game.

The HFHM game was developed according to the real-life context of the participating school, where each class was allocated a planting area. A puzzle (Figure 3) was designed to represent this scenario. Children in the experimental group were randomly divided into groups of four to cooperate in completing diet- and nutrition-related tasks to earn puzzle pieces. Rewards were distributed based on the order in which each group completed the puzzle, fostering a sense of healthy competition.

The game was implemented during lunchtime over a two-week period. In the first week, the daily tasks focused on encouraging positive dietary behaviors, including the following:

(1) Selecting food based on appetite and finishing the food you take to minimize waste.
(2) Trying all types of food served in the school lunch to minimize picky eating.
(3) Completing meals within the school’s designated time.

**Figure 4:**
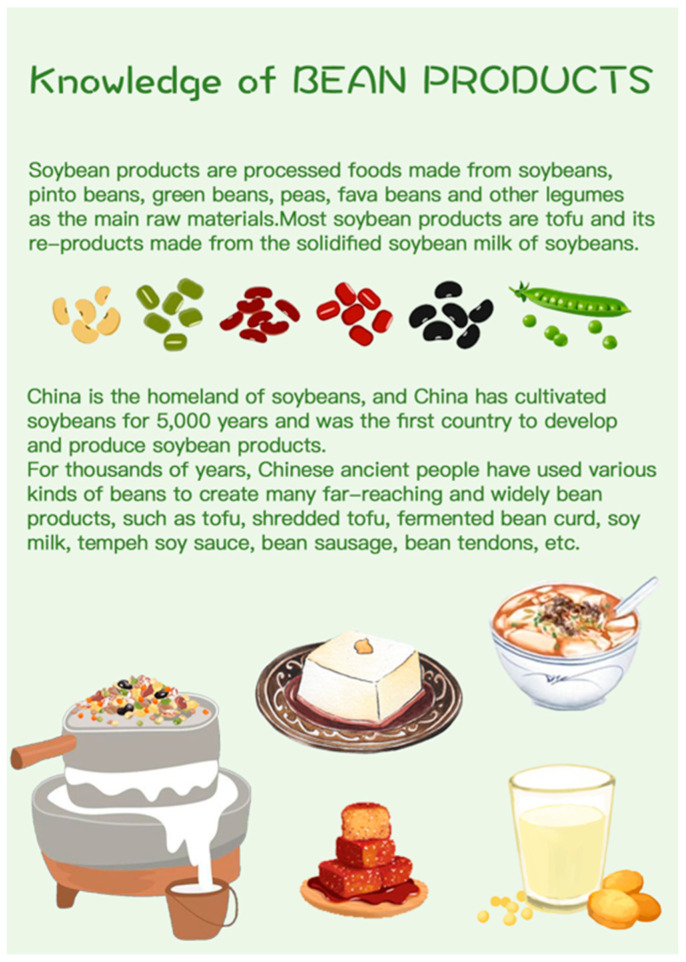
One of the knowledge cards. The original version of the card is written in Mandarin and utilizes simplified language designed to facilitate understanding for children.

In the second week, in addition to the above tasks, children in the intervention group participated in a five-question nutrition quiz each day, with questions drawn from the nutrition knowledge cards provided (Figure 4). These cards were designed according to the Dietary Guidelines for Chinese Residents [10] and introduced key information on essential nutrients and their roles in a healthy diet. Children were informed that they could take the cards home to learn the content with their parents. Additionally, they were also told that the content on the cards would be included as part of the game, promoting their active learning.

Children in the experimental group participated in the game daily during lunchtime, earning points by completing the aforementioned tasks. These points were then exchanged for puzzle pieces and nutrition knowledge cards. Researchers evaluated each group’s performance based on their eating behaviors and quiz results, with points awarded accordingly. Daily scores were posted on a leaderboard in the classroom to promote motivation and a sense of competition among groups.

At the conclusion of the two-week period, the group that completed their puzzle first was awarded the privilege of choosing and planting their preferred crop in their class’s designated garden area. Following the game, all children in the intervention group participated in a collective crop-planting activity. This final event marked the culmination of the intervention and reinforced the nutrition knowledge and healthy behaviors cultivated during the game.

In contrast, children in the control group continued their daily routines and lunchtime activities as usual, without participating in any intervention. Their lunch arrangements and daily schedules remained unchanged throughout the study. The control group also had a school-allocated plot, but their planting area was not linked to lunch activities; they just followed the school’s general arrangements for planting activities. Nutrition knowledge cards were distributed to the control group after the baseline data had been collected. However, they were not required to learn the content. This measure ensured that any differences observed could be attributed to the intervention provided to the experimental group.

### 2.5. Data Analysis

Data were analyzed using R software (vision 4.4.0). A *p*-value of <0.05 was considered statistically significant. Descriptive statistics, including means and standard deviations, were calculated to summarize the children’s eating behaviors. The distributions of the covariates gender, BMI, child’s age, caregivers’ education level, and family annual income at baseline between the control group and the experimental group were compared using the simulation-based chi-squared test. For each of the four outcomes (Knowledge, Food Waste, Meal Duration and Picky Eating), a mixed-effects linear model for the dependence on group and time adjusted for BMI, age, and gender was fitted using the lme4 and lmerTest packages in R software. Afterwards, a two-way rANOVA was conducted for each of the four outcomes. The assumptions of normality and homoscedasticity of the residuals were assessed visually via diagnostic plots. The *p*-values for the difference-in-differences as well as for the within-group changes were obtained from that model.

## 3. Results

### 3.1. Pre-Intervention Survey and Interview Results

Descriptive statistics from eating-behaviors questionnaires completed by caregivers (valid *n* = 38) are shown in Table 3. The most prominent problematic behaviors identified were picky eating (M = 2.76, SD = 0.47) and bad habits (M = 2.41, SD = 0.47).

The findings align with challenges identified in the semi-structured interviews, which highlighted specific issues with eating behavior, including picky eating, food waste, and slow eating. Four caregivers reported significant difficulties in encouraging their children to try new or varied foods, which often resulted in a limited diet that lacked variety. Furthermore, both caregivers and teachers observed that some children struggled to maintain focus during meals, leading to slower eating. This issue was particularly evident during school lunches, when some children were unable to finish their meals within the allocated time. In addition, food waste was identified as a recurring concern by both caregivers and teachers. This issue primarily stemmed from two factors: (a) picky eating, where children discarded disliked items, and (b) slow eating, which prevented children from completing their meals on time at school.

The distribution of the covariates at baseline are shown in Table 4. There were no statistically significant differences between the groups, implying successful randomization. None of the covariates (gender, BMI, age, caregivers’ education level, and caregivers’ annual income) was found to have a statistically significant effect either on the response level in general or on the difference between the groups (i.e., there was no modifying effect). They were thus omitted from further analysis.

### 3.2. Changes in the Experimental Group and Post-Intervention

The sample statistics, as shown in Table 5, the evaluated Cohen’s d, the estimated difference-in-differences effect, and the corresponding 95% confidence intervals (CIs) are shown in Table 2. All the differences-in-differences were found to be statistically significant at the 5% level.

**Figure 5:**
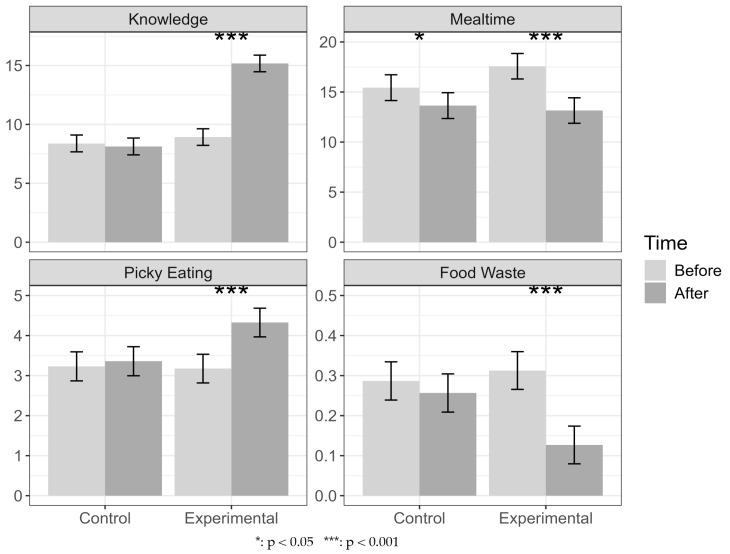
The estimated mean levels and 95% CIs by outcome and group, before and after the intervention.

The estimated mean levels and 95% CIs by outcome and group, before and after the intervention, are shown in Figure 5. The changes were highly statistically significant (*p* < 0.0001) in the experimental group for all four outcomes. In the control group, the only statistically significant change occurred in Meal Duration (*p* = 0.0335).

## 4. Discussion

This study evaluated the effects of Happy Farm, Happy Meal (HFHM), a school-context-based alternate reality game (ARG), on improving elementary children’s healthy eating behaviors and nutritional knowledge in China. To the best of our knowledge, this is the first paper to employ an ARG approach to promote learning about nutrition and changes in eating behaviors among Chinese elementary children. Our results show that children’s scores on nutrition-knowledge quizzes and three eating behaviors were significantly improved between the pre-test and the post-test assessments within the intervention group. In contrast, the performance of the control group was not significantly different between the pre- and post-test assessments. These results indicate that HFHM can serve as an effective tool for enhancing children’s nutrition education and promoting some changes in eating behavior. This finding is consistent with those of previous studies suggesting that gamified learning tools can enhance engagement, motivation, knowledge retention, and behavior change in children’s nutrition education [16,34,35]. Specifically, HFHM integrates real-world activities with interactive game mechanics, allowing children to actively engage in healthy eating habits and learn nutrition concepts as they apply them in daily life.

Previous studies have suggested that digital serious games and mobile applications can effectively enhance children’s nutrition knowledge and food choices [36,37,38]. However, most of these interventions rely on digital devices, including computers, tablets, and mobile phones, which may raise concerns among parents and teachers. Studies have emphasized that excessive screen time leads to increased risk of visual impairment, reduced physical activity, and potential addictive behaviors [39,40]. Additionally, in many Chinese elementary schools, the use of digital devices in non-academic settings is strictly regulated or discouraged, limiting the applicability of device-based nutrition-education programs [20].

In contrast, HFHM uses the principles of ARG to integrate nutrition education and eating-behavior changes into children’s real-life school environment without relying on any digital devices. In contrast to video games, ARGs embed educational content into children’s daily activities, making learning more contextualized, socially interactive, and directly applicable to real-world situations [41]. With real-world tasks, peer collaboration, and school-based scenarios, HFHM is an ideal tool for use within China’s education system, where group activities and structured learning are emphasized by both schools and families [42]. This could explain its suitability for this context and effectiveness in fostering behavioral changes and knowledge learning in Chinese children. Currently, school gardening programs are being adopted across China in increasing numbers [43]; schools with similar planting-area configurations can make targeted modifications to the HFHM framework, tailoring the game scenarios to better align with their specific contexts. By integrating this approach, schools can effectively utilize games like HFHM to influence children’s eating behaviors and enhance learning about nutrition. This adaptation allows for a more contextually relevant application of HFHM in different schools, which could potentially improve its efficacy among students from different areas and cultures. Schools can modify the HFHM game slightly to make the game more relevant to their contexts, applying it to effect changes in children’s eating behaviors and support nutrition education.

Furthermore, previous serious games targeting children’s nutrition education have mainly focused on food selection and consumption (e.g., increasing fruit and vegetable intake) [44,45], while HFHM aims at addressing a broader set of eating behaviors, including those related to meal duration, picky eating, and food waste. All these behaviors were identified via pre-intervention interviews and questionnaires, guaranteeing the suitability of the game to its audience. This highlights the potential for expanding game-based interventions beyond just food-choice education to incorporate holistic eating behaviors.

While this study provides valuable insights into Chinese children’s health education, the following main limitations are noteworthy.

First is the limited sample size and short study duration. As a pilot study, this experiment had a relatively small sample size and a short intervention period. Only 79 children from two classes participated, and the intervention lasted for just 10 days. While the findings provide initial evidence of the game’s effectiveness, the limited duration may not fully capture the long-term impact of HFHM on children’s nutrition knowledge and eating behaviors. Future studies should involve larger and more diverse participant groups and extend the experiment duration to assess the long-term effects of the ARG intervention.

Another limitation is the measurement of children’s nutrition knowledge. Since no standardized nutrition-knowledge quiz specifically designed for Chinese children was available at the time of the study, we developed a customized questionnaire with input from experts in nutrition and education. However, this instrument has not undergone formal validity and reliability testing, which may affect the accuracy of the results. Future research should use a validated nutrition-knowledge assessment tool tailored to Chinese children to ensure more robust and reliable measurements.

This study provides evidence that interventions based on alternate reality games can be alternatives to digital serious games in China’s schools. With the growing interest in game-based learning, future research could explore interventions that combine the interactive nature of ARGs with digital elements, such as mobile recording, to enhance engagement while mitigating concerns related to screen time. Additionally, further investigation is needed to assess the scalability and adaptability of HFHM across different educational and cultural contexts in China. Future studies should also examine the long-term effects of ARGs interventions. Lastly, collaboration with educators, policymakers, and nutritionists will be crucial in refining and expanding game-based interventions to better support children’s health education in school curricula.

## 5. Conclusions

This study demonstrated that Happy Farm, Happy Meal (HFHM), an alternate reality game (ARG), is a promising approach by which to engage Chinese school-aged children in nutrition education and promote healthy eating behaviors. Future research could explore the long-term effects of ARGs on dietary behavior and retention of nutrition knowledge, as well as their adaptability to other health-education contexts. Integrating ARGs into school curricula as enrichment activities can provide interactive and experiential learning experiences, reinforcing sustainable health practices from an early age. These findings highlight the potential of ARGs as innovative and effective tools for children’s nutrition education and for broader health-promotion initiatives.

## Figures and Tables

**Table 1:** Questions from semi-structured interviews.

Interview Topics	Example Questions (Caregivers)	Example Questions (Teachers)
Meal Patterns	What types of meals do you typically prepare for your child?	What types of lunch does the school typically prepare for students?
Children’s Eating Behaviors	What are your child’s favorite foods? Are there foods they refuse to eat?	According to your observations, do students show a preference for different foods? Can they refuse to eat foods they don’t like?
Barriers to Healthy Eating	What challenges do you face when trying to ensure your child eats a balanced diet?	What challenges do you face when trying to ensure students eat a balanced diet?
Nutritional Knowledge	How well does your child understand the concept of healthy eating and nutrition?	How well do students understand the concept of healthy eating and nutrition?

**Table 2:** Examples of questions from the questionnaire on nutrition knowledge.

Questions	Answer Options
According to the “Chinese Dietary Guidelines”, which type of food should form the main part of our daily diet? (Single choice)	A. Grains B. Dairy products C. Meat, poultry, and eggs D. Fruits and vegetables
According to the Chinese Dietary Plate, which food groups should be included in our daily nutritional intake? (Multiple choice)	A. Grains and tubers B. Fish, meat, eggs, and beans C. Vegetables D. Fruits

**Table 3:** Descriptive statistics summarizing children’s eating behaviors.

Characteristics	Means	SD
Picky	2.76	0.47
Reaction	2.40	0.58
Bad Habits	2.41	0.47
Overfull	2.69	0.43
External	2.42	0.62
Emotional	1.63	0.53
Active	2.62	0.45

The questionnaire used a five-point Likert scale, with reverse coding applied for the ACTIVE items. Higher scores indicate more significant problems in eating behavior, as reported by the child’s parents.

**Table 4:** Baseline demographics of all children in the two groups.

Characteristics	Experimental Group (*n* = 40)	Control Group (*n* = 39)	*p* Value
Gender			0.724
Male	21 (52.5%)	23 (59.0%)	
Female	19 (47.5%)	16 (41.0%)	
BMI ^1^			0.949
Underweight	5 (10%)	3 (7.7%)	
Healthy weight	28 (70%)	28 (71.8%)	
Overweight	4 (10%)	5 (12.8%)	
Obese	3 (7.5%)	3 (7.7%)	
Age			1.000
8.5	15 (37.5%)	14 (35.9%)	
9	25 (62.5%)	25 (64.1%)	
Caregivers’ education level			0.805
Junior school or below	14 (35%)	16 (41.0%)	
High school	18 (45%)	17 (43.6%)	
Associate/Bachelor or above	8 (20%)	6 (15.4%)	
Annual family income			0.448
≤¥40,000	-	-	
¥40,000–¥80,000	11	13	
¥80,000–¥120,000	20	15	
>¥120,000	9	11	

^1^ BMI: Body mass index.

**Table 5:** The sample statistics, the evaluated Cohen’s d, the estimated difference-in-differences effect, and the corresponding 95% confidence intervals for the study outcomes. The estimated effects, 95% CIs, and *p*-values are based on the two-way rANOVA.

Outcome	Control (*n* = 39)		Experimental (*n* = 40)			Estimated		
	Baseline	Change	Baseline	Change	Cohen’s d	Effect	95% CI	*p*-Value
Knowledge	8.38	−0.26	8.93	6.25	−2.47	6.51	(5.33, 7.69)	<0.0001
	(2.06)	(2.23)	(2.35)	(2.98)				
Food Waste	0.29	−0.03	0.31	−0.17	0.5	−0.16	(−0.24, −0.07)	0.0004
	(0.15)	(0.19)	(0.16)	(0.22)				
Picky Eating	3.23	0.13	3.17	1.15	0.68	1.02	(0.32, 1.73)	0.0045
	(1.06)	(1.38)	(1.38)	(1.75)				
Meal Duration	15.44	−1.79	17.58	−4.42	−0.65	−2.63	(−4.98, −0.28)	0.0264
	(3.12)	(3.96)	(5.91)	(6.25)				

## Data Availability

The data used in this study are available from the submitting author upon request.
